# An Interregional, Transdisciplinary and Good Practice-Based Approach for Frailty: the Mind&Gait Project

**Published:** 2019-01-06

**Authors:** J Apóstolo, F Couto, E Bobrowicz-Campos, MA Dixe, J Ribeiro, M Braúna, T Camacho, R Santos-Rocha, P Parreira, A Cruz, C Malça, C Dantas, L Jegundo, L Marcelino, M Simões, M Almeida

**Affiliations:** 1 Nursing School of Coimbra; 2 The Health Sciences Research Unit: Nursing, Nursing School of Coimbra; 3 Centre for Innovative Care and Health Technology, Polytechnic Institute of Leiria; 4 Sport Sciences School of Rio Maior, Polytechnic Institute of Santarém; 5 Interdisciplinary Centre for the Study of Human Performance, Faculty of Human Kinetics, University of Lisbon; 6 Mechanical Engineering Department, Polytechnic Institute of Coimbra; 7 Caritas Diocesana de Coimbra; 8 Informatics Engineering Department, School of Technology and Management, Polytechnic Institute of Leiria; 9 Santa Casa da Misericórdia de Alcobaça

**Keywords:** ageing, frailty, cognitive stimulation, physical exercise, digital solutions, technology transfer

## Abstract

Social facilities such residential structures and day-centres increasingly seek integrated, structured, adapted, creative, dynamic and economic strategies to prevent frailty. The arising need of an aged and frail population requires innovative interventions and products to prevent cognitive and physical decline. The interregional MIND&GAIT project aims to promote independent living in frail older adults by improving cognition and gait ability by using assistive products. This transdisciplinary strategy within a 24-months period expects as project’ deliverables: i) a structured and good practice-based combined intervention (CI) consisting of a cognitive stimulation programme and a physical exercise programme; ii) an auto-blocking mechanism for rolling walkers with biofeedback acquisition (ABMRW); iii) a randomized clinical trial to assess CI′ effectiveness; and iv) a web-platform to be used as a repository that will support and disseminate the intervention materials, covering the action-line of translational research. Positive benefits are expected in prevention and maintenance of frail older adults’ capacities. Preliminary results showed positive effects on the improvement of cognitive and physical functions, functionality and depressive symptomatology. The interregional geographical coverage induced by MIND&GAIT underlines the potential replicability of the project extension to the community in the Centro and Alentejo regions of Portugal. MIND&GAIT network supports actions and provides learning opportunities and emergence of locally-embedded support systems towards social innovation for older adults.

## I. INTRODUCTION

### Socio-demographic context

Current Europe confronts with the global phenomenon of demographic ageing, considered as one of the greatest social and economic challenges of the 21st century [1]. In 2017, Portugal registered an ageing rate of 153.2% [2] and projections from the Organization for Economic Cooperation and Development [OECD] predict that by 2050, 40% of the resident population in the country will be 65 years or more [3]. There is an evident rise of geriatric comorbidities and, due to physical and cognitive decline, institutionalization of older adults has increased. In 2017, Portugal had a 32.9% older adults’ dependency rate, mainly in Alentejo (40.4%) and Centro (37.4%) regions, considered the most aged country regions [2].

To face this reality and to deal with older adults’ and their relative’s needs, worldwide action lines are being proposed to fairly guarantee geriatric healthcare accessibility, credibility and sustainability. Guidelines, policies, strategies and action plans, continually emerge focused on reflecting and give affordable and effective solutions for older adults needs, in terms of well-being and quality of life [4][5]. This worldwide approach, supported by the World Health Organization (WHO) [6] is reflected in the European context through action plans, such defined by the European Innovation Partnership for Active and Healthy Ageing (EIPAHA) [7][8] that noticeably marks the need of enhancing physical and cognitive decline, as key points to promote older adults health [9][10].

One of the most controversial and worrisome conditions associated with global ageing is frailty [11]. As an age-related state of decreased physiological reserves, frailty is characterized by a weakened response to stressors associated with an increased risk of adverse health-related outcomes [11]. This clinical condition nettles a continuous decline in functional domains and enhances the risk of geriatric syndromes’ development [12].

Preventing frailty’s evolution is an emerging priority [4] due to its high prevalence and negative effects. As a vulnerable population, frail older adults, must be empowered and should receive support from different health professionals integrated into transdisciplinary teams [13]. These teams should work on the improvement of independent living to promote healthcare quality [11][14][5]. Geriatric transdisciplinary research projects can work towards the improvement and management of health-related outcomes based on the identification of priorities, real needs, and available resources [4].

Evidence-based practice constantly generates new challenges and research questions that should reflect real needs of frail older adults. Innovative procedures from evidence-based practice are required as their needs and priorities are changing, especially considering the context of increasing technological society.

### Guidelines, strategies and policies

Traditionally, geriatric interventions that have been applied by healthcare professionals at a primary level adopt, mostly, a curative approach, instead of a proactive and preventive one. To face this reality MIND&GAIT aims to promote independent living of frail older adults by improving cognition through a combined intervention (CI) and also gait ability by using innovative assistive products.

MIND&GAIT is sustained by WHO guidelines [6], action lines from the EIPAHA [7][8], and also health national strategies [15]. In regard to more recent events, it is clear that MIND&GAIT also fits into the Lisbon Declaration [16] for social innovation as a path to a sustainable, resilient and inclusive Europe. Once, it answers to priority number five that is related to prioritizing social innovation’ spreading to regions where it is most needed by creating a network for regional support, connecting educational and health organizations at a local, regional and transregional levels that help to spread health and social innovations related to ageing healthcare.

### Interregional geographical coverage

Achieving universal health coverage means ensuring that all people receive the essential good practice-based on health services that they need without being exposed to financial hardship as a result [17]. By spreading social and health innovation in frailty to regions where it is most needed, this project is contributing to health equity. Portugal is one of the most aged countries in Europe. Considering the extended aged population and dependency rates of the Centro region of Portugal, MIND&GAIT arises as an opportunity to different research teams and stakeholders to work aligned for the development and implementation of good practices in ageing.

The consortium comprises four academic institutions from the Portuguese cities of Coimbra, Leiria and Rio Maior and two social care’ end-user organizations from Coimbra and Alcobaça. MIND&GAIT encourages a network-building for more active and sustainable involvement of organizations and end-users in promoting older adults’ health and well-being. The network allows partners from different regions to make better use of the existing endogenous potentials of a diverse academic workforce by awareness-raising on demographic change, developing ageing strategies as well as state-of-the-art training concepts. The interregional geographical coverage induced by MIND&GAIT, also underlines the potential replicability of community’ project extension in Centro and Alentejo regions.

## II. METHODS

MIND&GAIT emerged as a response to societal challenges and targets older adults with 65 years or more that are supported by the consortium end-user organizations, who are frail or at risk of developing frailty.

By adopting a transdisciplinary approach, the research team followed a methodology framework within a cross-disciplinary and problem-oriented research. The project proposed an integrated, creative and dynamic strategy to prevent frailty with a multicentric and a co-designed methodology. MIND&GAIT, based on team expertise, developed and tested interventions and products to promote independent living of frail older adults by improving cognition and gait ability

The project, conducted by a transdisciplinary research team, has developed seven working-packages (WP) in a 24-months period. It involves the scientific areas of nursing, occupational and physical therapy, psychology, exercise, mechanical and computer engineering and social care. To ensure the best solutions for frailty through the congregation of different types of expertise and “know-how”, each scientific area established specific objectives adopting adequate scientific methods. For each WP a specific objective was defined to:

- Develop and test a cognitive stimulation programme to prevent psychological frailty (WP1);
- Develop and test a physical exercise programme to prevent physical frailty (WP2);
- Develop an auto-blocking kit mechanism for rolling-walkers and to instrument it with pressure and balance sensors and also with educational led lights (ABMRW) to prevent and reduce falls (WP3/4);
- Assess the effectiveness of CI in older adult’s frailty, composed by cognitive stimulation and physical exercise programs (WP5);
- Develop a web-platform to support healthcare professionals and to disseminate CI materials’ and its implementation process (WP6);
- Analyze participant perception of the interventions and products developed (WP7);

The experts from nursing, occupational therapy, psychology and exercise worked on the development and assessment of two programmes that were expected for WP1 and WP2. The methodological approach was established under the guidelines for complex interventions’ development from The Medical Research Council [18]. For both programmes, the development process comprised four phases. In first phase - Preliminary Phase (I) – through focus groups an initial programmes design’ conceptualization and its supporting materials was developed. Students from nursing, occupational therapy and sports were also included and contributed to programs’ elaboration. The second phase - Modelling Phase (II) - gathered different opinions and evaluations from specialists in the area of cognitive and physical exercise interventions through an experts’ panel. The third phase - Field Test Phase (III) - all sessions of the programs were evaluated and validated. The final phase - Consensus Conference Phase (IV) - synthesized all contributions and analyses resulted from the previous phases. In addition, WP1 also involved the scientific area of informatics engineering, once it was expected a digital transformation of the cognitive stimulation program.

An enhanced methodological approach was adopted on WP3 and WP4. The upgrade of an ABMRW, and also the integration of technological and interactive components to provide biofeedback parameters acquisition (WP3) were developed under orientation of the scientific areas of mechanical and electromechanical engineering and also rehabilitation nursing. Two self-locking alternative single mechanical approaches were developed to be upgraded in a new rolling-walker or to be applied on existing ones: i) using a set of gears and a spring; and ii) using a single spring. In addition, to ensure biofeedback parameters acquisition and to promote the interaction between the user and the rolling-walker, pressure and balance sensors and also educational led lights were applied. The main objective was to prevent older adults’ falls. WP4 comprehended the test of the rolling-walker with the system that was upgraded in WP3. For the WP purpose, two quasi-experimental studies (pre-post design, single group), with non-probability sample obtained by convenience of older adults that normally use walkers, were carried out.

After the two programmes (WP1 and 2) being develop and validated, were unified in order to form CI. CI′ effectiveness was assessed in WP5 through a randomized controlled trial. The primary outcome measures of the study were: cognitive function – assessed with *Montreal Cognitive Assessment (MoCA)* [19][20], gait speed [21], risk of fall based on gait and balance – assessed with *Tinetti Index* [22][23], biomechanical parameters of gait – assessed with *NovelEMED-X pressure platform* in accordance with a protocol designed for purpose and based on the directives of [24], and depressive symptomatology - assessed with *Geriatric Depression Scale 10* [25][26]. Activities of daily living - assessed with *Barthel Index* [27][28] used with the permission of Barthel Index Portugal/European Portuguese Mapi Institute - was secondary outcome measure.

For WP6, researchers from all scientific areas were involved and participated in a focus group to gather different opinions of how the web-platform should be structured and what type of contents should be there reflected. The platform and its software are being developed by the scientific area of informatics engineering.

WP7 is transversal to all WPs. Focus group and semi-structured interviews were made in order to analyze participant’s perception.

## III. EXPECTED OUTCOMES

Based on the proposed methodology and WPs several deliverables and intervention products culturally validated and adapted are expected:

- A digital cognitive stimulation programme available from a mobile application and a web-platform. This will provide individual and group sessions of cognitive stimulation;
- An *ebook* containing the physical exercise program for frail older adults. A portfolio of exercises will provide guidelines on providing functional training sessions;
- An ABMRW associated with technological and interactive components that will provide biofeedback parameters acquisition, contributing to the safety of frail older adults by preventing and reducing falls;
- A web-platform for knowledge exchange working as a repository. This tool will guide end-users, health professionals and formal/informal caregivers on the CI implementation. It will also contribute to good practice replication covering the action-line of translational research, by empowering citizens for the ageing process.
- Academic research collaborations and multi-stakeholder partnerships;
- Citizen engagement through the inclusion of older adults and caregivers during the research process by the evaluation of feasibility, appropriateness and meaningfulness of the developed strategies and interventions.
- Practical orientations based on science translation that guide and support health and social care professionals towards independence of frail older adults.

Nearly 100 older adults have already beneficiated from the products developed within the project framework. Positive benefits are expected in the prevention and maintenance of physical and cognitive decline of frail older adults. It is expected that the developed products bring autonomy and quality of life’ improvement, contributing to active, healthy and participatory ageing.

CI preliminary results showed positive effects on the improvement of cognitive (namely in the orientation neurocognitive domain) and physical functions (namely in walking speed and balance), functionality, and also depressive symptomatology’ reduction. CI also fostered enjoyable moments in an age-friendly environment that triggers new forms of learning tailored to each specific context.

## IV. DISCUSSION

Health system’ functioning relies essentially on access to affordable and necessary interventions of assured quality, that are available at a primary healthcare level [17]. The implementation of multicomponent programmes carried out at the primary healthcare level is essential to prevent and manage all adverse health-related outcomes that are associated to the ageing process and specifically to frailty [1][6].

Many are the advantages that are pointed out for cognitive and physical interventions. When physical and cognitive impairment, depressive symptomatology and pain are understood as inevitable consequences of ageing, older adults are unlikely to be included as an active citizen and may become marginalized, particularly in societies where independence and self-sufficiency are highly valued [6]. The solution goes through implementing multidomain and preventive nonpharmacological interventions that are proven to be more effective in frailty context [11][17][29], including physical activity or exercise, cognitive stimulation, healthy dietary habits and promotion of resilience [30]. Good practices link real needs with the best scientific evidence and facilitate knowledge transference to clinical practice. Future generations would benefit if health interventions were interconnected with digital solutions, which encourage older adults and their caregivers to act and intervene more in their health maximizing their functional capacity [31].

Older adults recognize that technology can have benefits for their independence and well-being, decreasing the impact of declining capacities [32]. The digital solutions that are proposed by MIND&GAIT (i.e. digital cognitive stimulation program, physical exercise program *ebook* and web-platform) could be used as non-pharmacological interventions to prevent or reduce frailty.

All products derived from a synthesis and convergence of several scientific languages, which constantly induces an adaptation and standardization of the different geriatric approaches, allowing the development of accessible supporting materials. So far, many of end-user organizations already offer digital solutions and devices that can be used for CI′ implementation. This good practice can then be applied by nurses, occupational therapists, psychologists, social care and other health professionals, and also exercise specialists and formal/informal caregivers.

The web-platform will disseminate CI and its components, reducing regional disparities of care and the ageism’ tendency, by providing to everyone the possibility of exchanging knowledge on practices between urban and rural areas, at regional and national levels.

Health professionals, researchers, policymakers and other stakeholders will be able to know about and to have access to the developed intervention and products, increasing their dissemination and replication.

Due to the high number of involved partners, the opportunity for broadening the contact network is real and supports the effective potential of transferability, so that it can be made available to other professionals and older adults. The scientific network is also a guarantee of project scalability.

All developed products reflect economic and environmental sustainability underlined by the opportunity of transferability and replicability, which is associated with their non-pharmacological nature and low cost.

Daily clinical practice can thus be effective and efficient if the knowledge, developed products, and respective achieved scientific results, influence new health policies that actively contribute to the societal challenge to which the project responds.

By minimizing negative effects and impacts of demographic trends on an ageing society, MIND&GAIT could improve existing framework conditions by adapting policies, governance processes and mechanisms.

## V. CONCLUSION

Geriatric social facilities increasingly seek integrated, structured, adapted, creative, dynamic and sustainable strategies to prevent frailty. MIND&GAIT showed to be an adequate strategy and opportunity to develop products that maximize and potentiate older adults’ physical and cognitive functions, through a proactive and preventive approach.

Preserving functionality and reducing comorbidities associated with age-related declines are important parts of healthy, active, participatory and successful ageing policies and care. The project, being based and tested in clinical practice, will guide health professionals, caregivers and public to promote independence of this population.

The MIND&GAIT network will support actions and provide learning opportunities and emergence of locally-embedded support systems for social innovation. By adopting a good practice-based approach, the quality of services and care provided is reinforced, contributing for intra and interregional dissemination and replication. The social and digital innovations that characterize the project could be understood as a path to a sustainable, resilient and inclusive society, first at a local and regional level and then, at a national level.

The project fostered transdisciplinary academic and stakeholders partnerships that translate into well-founded and real gains in health. It praises the necessity to understand healthy, active and participatory ageing as a shared social responsibility, through awareness-raising for more inclusive, innovative and reflective societies, contributing to the general culture of health.
